# Patterns of failure for glioblastoma multiforme following limited-margin radiation and concurrent temozolomide

**DOI:** 10.1186/1748-717X-9-130

**Published:** 2014-06-06

**Authors:** Brian J Gebhardt, Michael C Dobelbower, William H Ennis, Asim K Bag, James M Markert, John B Fiveash

**Affiliations:** 1 University of Texas Southwestern-Austin, 601 E. 15th St, Austin, TX 78701, USA; 2 University of Alabama at Birmingham, 1700 6th Ave. South, Birmingham, AL 35233, USA

**Keywords:** Glioblastoma, Radiotherapy, Margin, Patterns of failure

## Abstract

**Background:**

To analyze patterns of failure in patients with glioblastoma multiforme (GBM) treated with limited-margin radiation therapy and concurrent temozolomide. We hypothesize that patients treated with margins in accordance with Adult Brain Tumor Consortium guidelines (ABTC) will demonstrate patterns of failure consistent with previous series of patients treated with 2–3 cm margins.

**Methods:**

A retrospective review was performed of patients treated at the University of Alabama at Birmingham for GBM between 2000 and 2011. Ninety-five patients with biopsy-proven disease and documented disease progression after treatment were analyzed. The initial planning target volume includes the T1-enhancing tumor and surrounding edema plus a 1 cm margin. The boost planning target volume includes the T1-enhancing tumor plus a 1 cm margin. The tumors were classified as in-field, marginal, or distant if greater than 80%, 20-80%, or less than 20% of the recurrent volume fell within the 95% isodose line, respectively.

**Results:**

The median progression-free survival from the time of diagnosis to documented failure was 8 months (range 3–46). Of the 95 documented recurrences, 77 patients (81%) had an in-field component of treatment failure, 6 (6%) had a marginal component, and 27 (28%) had a distant component. Sixty-three patients (66%) demonstrated in-field only recurrence.

**Conclusions:**

The low rate of marginal recurrence suggests that wider margins would have little impact on the pattern of failure, validating the use of limited margins in accordance ABTC guidelines.

## Introduction

More than one-third of the approximately 67,000 cases of primary brain and other nervous system tumors diagnosed each year in U.S. adults are malignant. GBM is the most common malignant histology and represents a disproportionate cause of cancer morbidity and mortality, even though primary brain tumors account for only 2 percent of all cancers [1]. Despite significant therapeutic advances in recent years, the prognosis of GBM remains dismal, with few patients surviving beyond 5 years. The addition of temozolomide resulted in an improvement in median survival from 12.1 to 14.6 months [2].

Studies comparing whole-brain with partial-brain irradiation showed no benefit from whole-brain irradiation, leading to the current practice of partial-brain treatment [3]. Autopsy series have shown that the pattern of failure of GBM after radiation therapy is predominantly within 2- to 3-cm of the primary tumor bed, which has led to the adoption of 2- to 3-cm radiation margins in conventional protocols [4-6]. The volume of irradiated brain is associated with the development of neurotoxicity [7,8]; therefore reducing the treatment volume may ameliorate these side effects. The margins defined by the ABTC are smaller than those utilized in protocols from the Radiation Therapy Oncology Group (RTOG) and European Organization for Research and Treatment of Cancer (EORTC). We hypothesize that patients treated in accordance with ABTC guidelines will demonstrate patterns of failure consistent with previous series of patients treated with 2–3 cm margins.

## Materials and methods

### Selection of patients

This retrospective study was approved by the University of Alabama at Birmingham (UAB) Institutional Review Board. The records of all patients treated for GBM at UAB between April 2000 and November 2011 were retrospectively assessed. This review included only patients with biopsy-proven GBM who suffered documented disease progression following treatment with radiation therapy and concurrent temozolomide. Adequate imaging prior to treatment, radiation dosimetry records, and radiographic assessment at failure were available for all patients included in this review.

### Treatment

All patients were treated at UAB according to the ABTC guidelines for radiation oncology. The most recent version of these guidelines is shown in Table 1. The prescribed dose varied minimally and generally was 46 Gy to an initial gross tumor volume (GTV) encompassing the primary tumor and surrounding edema on post-operative T2 or FLAIR MRI. This was expanded by 5 mm and then edited to conform to anatomic barriers to tumor spread in order to create the initial clinical target volume (CTV). The CTV was expanded an additional 5 mm to generate the initial planning target volume (PTV). The boost GTV was defined as the residual T1 contrast-enhancing tumor plus resection cavity and was expanded in similar fashion. The boost PTV was prescribed an additional 14 Gy, and the total dose to the boost volume was 60 Gy delivered in daily fractions of 2 Gy. The dose-reference point was the International Commission on Radiation Units (ICRU) reference point, usually the isocenter located in the center of the boost volume. Radiotherapy plans were normalized so the 95% isodose line encompassed the PTV completely.

**Table 1:** ABTC guidelines for target definition

**Target volume**	**Definition**
GTV1	T1 enhancing and non-enhancing tumor volume (T2 or FLAIR)
GTV2	T1 enhancing tumor volume
CTV1;2	GTV plus a margin of 5 mm
PTV1;2	CTV plus a margin of 3–5 mm

Note: GTV volume is based on postoperative day 0–1 MRI. GTV, Gross Tumor Volume; CTV, Clinical Target Volume; PTV, planning target volume; ABTC, Adult Brain Tumors Consortium.

Patients were followed with serial MRI scans at 1 month post-radiation and then at 2-month intervals. Typical imaging sequences included pre- and post-contrast T1, T2, and FLAIR. Newer techniques including perfusion, diffusion, and MR spectroscopy were used in follow-up of many patients beginning in 2005. Because of the possibility of post-radiation imaging changes or pseudo-progression, an imaging or clinical change developing after radiation in the absence of recurrent tumor, changes at the first follow-up scan were managed conservatively with observation or a trial of steroids. Patients outside of this time frame or refractory to steroid therapy were generally considered to have progressive disease, and additional treatment was at the discretion of the treating physician.

### Analysis

The determination of disease progression was made by the treating physician with the help of the UAB Central Nervous System (CNS) multidisciplinary tumor board based upon clinical and radiographic changes. MRI scans documenting failure after concurrent temozolomide and radiation were fused to the original treatment planning CT scans electronically using the ECLIPSE treatment planning system (Varian Medical Systems, Palo Alto, CA, USA). In order to reduce bias, the recurrent tumor volumes were generated by contouring the contrast-enhancing abnormalities on the MRIs prior to fusion with the isodose curves of the treatment plan. The recurrent tumors were analyzed to determine the volume of recurrent tumor present within the 95% isodose line of the boost plan of the completed treatment. The recurrent tumors were classified as “in-field” if >80% of the T1-enhancing tumor volume was covered by the 95% isodose line, “marginal” if >20 but ≤80% of the tumor volume was within the 95% isodose line, or distant in <20% of the tumor volume was located within the 95% isodose line. In cases of multiple discrete sites of failure, each lesion was independently analyzed relative to the 95% isodose line. Follow-up time was calculated from the date of pathological diagnosis of GBM until the most recent follow-up visit with imaging.

### Statistics

The Kaplan-Meier method was used to estimate the rates of time to progression. Associations of patient factors with time to progression were assessed with the log-rank test. Statistical significance was determined at the 5% level. Analyses were conducted using SPSS, version 21.

## Results

### Patient characteristics

Ninety-five cases of recurrent GBM initially treated with concurrent temozolomide and radiation therapy between April 2000 and November 2011 with adequate imaging at time of failure and sufficient data for reconstruction of radiation dose distributions were identified and included in the analysis. Of the 90 patients for which surgical data was available, 27 patients (30%) had undergone a gross total resection, 50 patients (56%) had undergone a subtotal resection, and 13 patients (14%) had only been biopsied.

Ninety-two of the 95 patients received 60 Gy in 30 fractions, 1 patient received 59.4 Gy in 33 fractions, and 2 patients received 56 Gy in 28 fractions. In all cases, patients received daily temozolomide at 75 mg/m^2^ concurrent with radiation. Additional temozolomide was given adjuvantly in 83 of 95 patients, delivered at a dose of 150–200 mg/m^2^ on each of the first 5 days of every 28-day cycle. Patients received a median of 6 (range, 0–26) cycles of adjuvant temozolomide. Patient and treatment characteristics are summarized in Table 2.

**Table 2:** Patient and treatment characteristics (n = 95)

**Feature**	**Number**	**Percentage**
Sex		
Male	52	55%
Female	43	45%
Age		
Median	55	
Range	19-77	
Tumor		
Unifocal	85	89%
Multifocal	10	11%
Surgery		
Biopsy only	13	14%
STR	50	56%
GTR	27	30%
Radiation		
60 Gy	92	97%
<60 Gy	3	3%
Adjuvant TMZ		
Concurrent TMZ	95	100%
Received post-RT TMZ	83	87%
Did not receive post-RT TMZ	12	13%
Median cycles	6	
Range of cycles	0-26	

*Abbreviations:GTR* Gross tumor resection, *STR* sub-total resection, *TMZ* temozolomide.

Twenty-eight patients (29%) were treated as part of an ABTC clinical trial and received additional trial drugs. Thirteen patients received Cilengitide, 9 patients received ABT-510, 2 patients received AT-101, and 4 patients were treated with hydroxychloroquine.

### Outcomes

**Figure 1:**
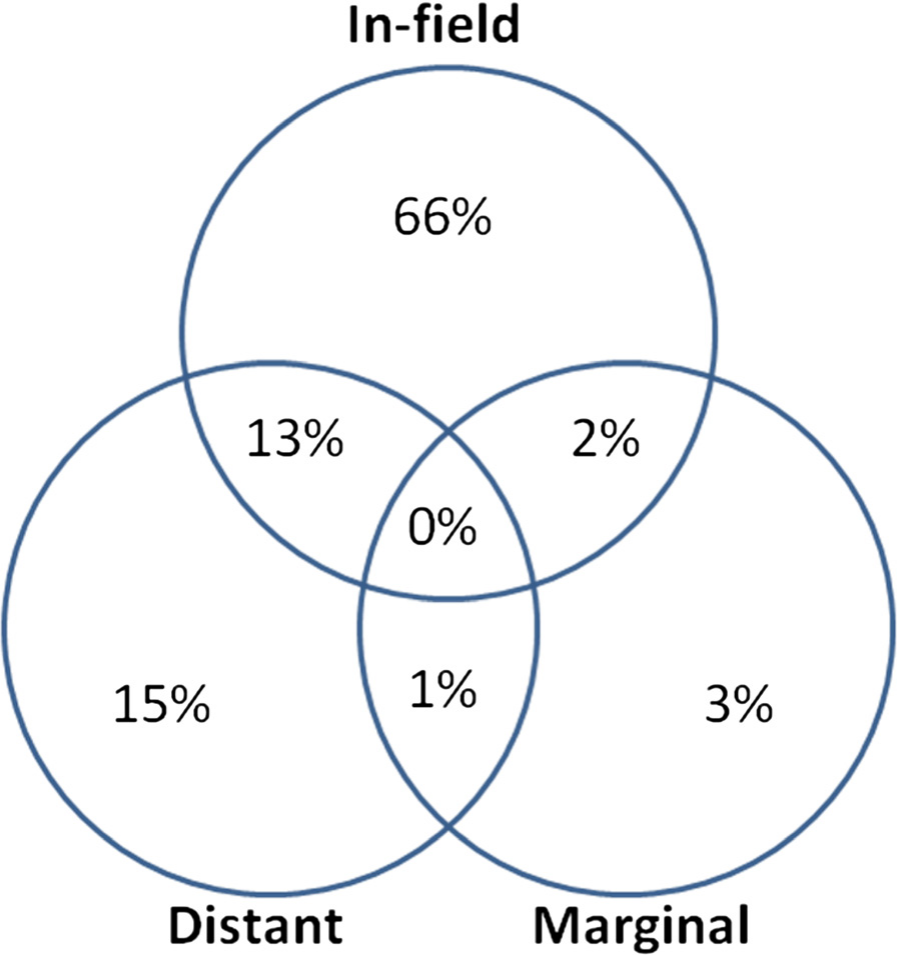
Patterns of Failure.

**Figure 2:**
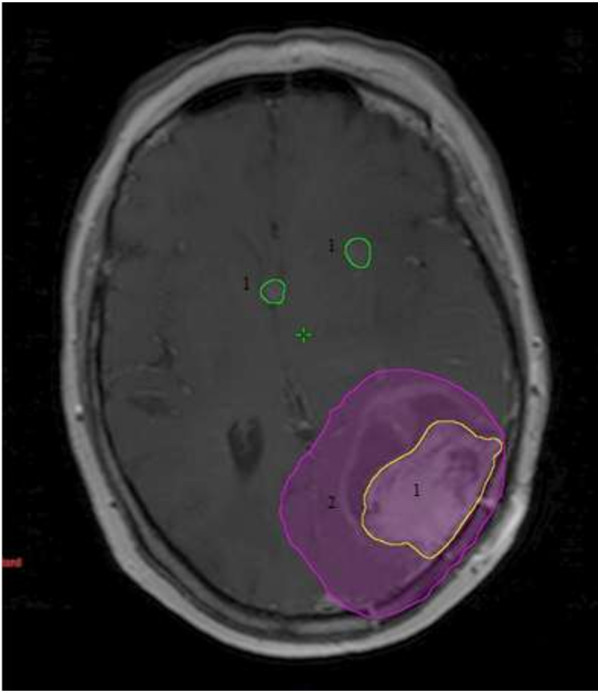
**Example of local and distant failure:** 1. sites of failure. 2. 95% isodose line of boost dose.

**Figure 3:**
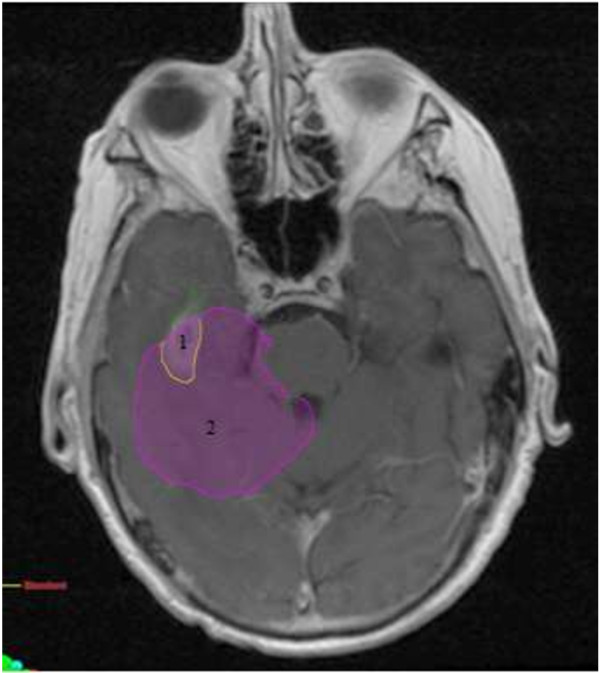
**Example of marginal failure:** 1. sites of failure. 2. 95% isodose line of boost dose.

The median time from completion of radiation to recurrence documented by MRI was 8 months (range, 3–46). The median volume of recurrent disease was 19.08 mL (range 0.11-234.11). The association between time to recurrence and location of failure was assessed. For the purpose of this analysis, sites of treatment failure were categorized into those containing no distant component of recurrence (i.e. in-field or marginal failure only) and those containing a distant component of recurrence. There was no association between time to recurrence and location of failure (p = 0.508). There was also no association found between type of resection (p = 0.285), or presence of multifocal disease (p = 0.233) and time to recurrence.The patterns of failure relative to the treated volumes are summarized in Figure 1. Of the 95 documented recurrences, 77 patients (81%) had an in-field component of treatment failure, 6 (6%) had a marginal component, and 27 (28%) had a distant component. Sixty-three patients (66%) demonstrated in-field only recurrence. Three (3%) failures were marginal only, 14 (15%) were distant only, 2 (2%) were in-field and marginal, 12 (13%) were in-field and distant, and 1 (1%) was marginal and distant. Of the 27 patients with a distant component of failure, 4 patients (15%) originally had multifocal disease. There was no association between pattern of failure and extent of resection or multifocal disease. Examples of distant and marginal failures are shown in Figures 2 and 3, respectively.

## Discussion

Our institution has previously reported our experience treating 20 patients with standard doses of radiation using limited margins with concurrent and adjuvant temozolomide from April 2000 to June 2005 [9]. The present analysis combines this data with those of an additional 75 patients treated from 2005 to November 2011. We found that the predominant pattern of failure was within the treatment volume, as 81% of patients had an in-field component to their disease progression. Six percent of patients had a marginal component to disease progression, and 28% had a site of failure distant to the treatment field. No factors were found to be associated with the pattern of failure or time to disease progression.

Autopsy studies have shown that glioblastoma patients treated with radiotherapy fail within 2- to 3-cm of the primary tumor bed, which has led to the use of analogous treatment margins. Several institutions have examined the pattern of failure following radiation therapy using conformal techniques with 2- to 3-cm margins and found that the pattern of failure remains predominantly within the treatment field [10-13].

A number of studies have looked at patients treated with radiation therapy prior to the introduction of temozolomide and found that the pattern of failure is predominantly local. A study by Lee reported failures among 36 patients treated with radiation alone to 70–80 Gy at the University of Michigan using 3D-conformal technique, and found that 89% of patients experienced failures within the treatment field. A follow-up publication by Chan examined an additional 34 patients treated with radiation alone. Despite dose escalation to 90 Gy, 91% still failed within the treatment field. All other patients experienced marginal failures, and no distant failures were observed in this series.

Another study of the patterns of failure of GBM after radiation and concurrent temozolomide was published by Brandes *et al.*[14]. In this study, 95 patients were treated with radiation, concurrent and adjuvant temozolomide. The patients received 60 Gy delivered to the T1 and T2 lesions on MRI with a 2- to 3-cm margin for the CTV with no field reduction during treatment. Despite using considerably larger target volumes than the present study, Brandes reported 21.5% of recurrences outside the radiation field, and 6.3% of recurrences at the margin of the radiation field. This study also found that 06-methylguanine DNA methyltransferase (MGMT) status was associated with the pattern of recurrence. Eighty-five percent of patients with unmethylated MGMT failed in-field or at the margin, compared with 57.9% of patients with methylated MGMT. Recurrences outside of the RT field occurred at a significantly longer time interval than those within the treatment field.

Glioblastoma research protocols typically employ radiation margins according to RTOG or EORTC guidelines. The EORTC uses a single-phase technique in which the GTV is defined as the surgical tumor bed plus any residual enhancing tumor. This is expanded by 2–3 cm to create the CTV and another 0.5-0.7 cm for the PTV. The GTV in RTOG protocols is similarly defined, but the CTV is created by including peritumoral edema. This is then expanded 2.0-2.5 cm to create the initial PTV. This initial volume is treated to 46 Gy in 23 fractions before a cone-down in which the GTV is expanded by 2.5-3.0 cm without accounting for edema. This final PTV is then treated with an additional 14 Gy. These guidelines are in contrast with ABTC guidelines in which the GTV is expanded 0.5 cm to create the CTV and another 0.5 cm for the PTV as described above.

Increasing the margin size can substantially increase the total volume of brain irradiated. Treating a round tumor with a 5 cm radius using a 1.0 cm total GTV to PTV margin will result in a total treatment volume of 452 cm^3^. If this same tumor is treated using a 2.5 or 3.5 cm total margin, then the resulting treatment volume is increased to 707 or 908 cm^3^, respectively. In this example, increasing the margin by 2.5 cm more than doubles the treatment volume, which would be expected to increase toxicity to the patient.

In recent years, several institutions have reported their experiences with limited-margin radiation in adherence to ABTC guidelines [9,15,16]. Brain irradiation is associated with neurotoxic side effects including radionecrosis and cognitive decline [7,8]. The volume of irradiated brain is believed to be associated with the development of these complications [17], and treating smaller volumes should theoretically reduce these effects. While the long-term prognosis of glioblastoma remains dismal [2], quality of life is an important consideration for patients treated with radiation.

Emory University published their experience with 43 patients with disease progression after treatment with concurrent temozolomide and radiation with 0.5 cm CTV margins [15]. They found that 93% of patients recurred within the treatment field, 5% were marginal, and 2% were distant relative to the 60 Gy isodose line. The researchers further analyzed the patients by creating hypothetical PTVs for each patient based upon RTOG guidelines. They found no difference in the distribution of pattern of failure when the two boost techniques were compared and concluded that a GTV to PTV margin of 1 cm or less did not appear to increase the risk of marginal or distant tumor failures.

A large series from Wake Forest examined 161 patients treated with radiation with 5-, 10, and 15- to 20-mm CTV margins [16]. Thirty-four patients were treated with 5-mm margins as in the present study. The researchers classified the patterns of failure as either within the 60 Gy volume, within the 46 Gy volume, marginal (within 2 cm from the 46 Gy volume), or distant (beyond 2 cm from the 46 Gy volume). They found no statistical difference between patients treated with different margins and the patterns of failure. There was no significant difference in progression-free survival or overall survival among the different treatment margin groups. Patients who failed within the 46 Gy volume had improved overall survival compared with patients who failed within the 60 Gy volume.

Chang *et al*. published a series that addressed the question of whether or not to include peritumoral edema in CTV delineation for glioblastoma [18]. They examined 48 patients who received three-dimensional conformal radiation with a 2-cm margin that did not include edema within the CTV and subsequently experienced disease progression. They generated hypothetical treatment plans according to RTOG guidelines that specify inclusion of peritumoral edema. Ninety percent of patients failed in central and in-field localization in either treatment plan. They found no statistical correlation between the location of recurrent tumor and edema volumes, and the pattern of failure was identical between the two sets of plans. Patients with volume of edema >75 cm^3^ would have 18% of their brain irradiated to 60 Gy under the RTOG guidelines compared with 7% when peritumoral edema was excluded.

The present study of 95 patients with disease progression after treatment with radiation therapy and concurrent temozolomide represents the largest series of patients treated with 0.5 cm CTV margins (1.0 cm total PTV margin). Acknowledged limitations of this study include its retrospective nature with inherent problems of selection bias. As described above, a subset of 28 patients was treated as part of an ABTC clinical trial and received additional trial drugs that may have influenced tumor cell activity and the patterns of disease progression. Another possible weakness involves the difficulty of distinguishing tumor progression from pseudoprogression, though we attempted to minimize the impact of this flaw by considering transient changes to represent pseudoprogression. The criteria from the Response Assessment in Neuro-oncology (RANO) Working Group were eventually adopted to evaluate disease progression, but the majority of patients were deemed to have progression prior to their publication. Despite these weaknesses, the data demonstrate a pattern of marginal failure consistent with previous studies using larger radiation margins.

## Conclusions

The use of limited-margin radiation therapy with concurrent and adjuvant temozolomide in the treatment of glioblastoma produces patterns of failure consistent with the existing literature. The low rate of marginal recurrence suggests that wider margins would have little impact on the pattern of failure, validating the use of limited margins in accordance with Adult Brain Tumor Consortium guidelines.

## Competing interests

The authors declare they have no conflicts of interest.

## Authors’ contributions

BG collected patient characteristics and treatment outcomes data, analyzed data, and drafted the manuscript. MD contributed to the design of the study and collected patient characteristics and treatment outcomes data. WE collected patient and treatment characteristics. AB participated in the care of patients treated in the study and helped to conceive of the study. JM participated in the care of patients treated in the study and helped to conceive of the study. JF participated in the care of patients treated in the study, contributed to the design of the study, and helped to draft the manuscript. All authors read and approved the final manuscript.
